# Experimental realization of an entanglement access network and secure multi-party computation

**DOI:** 10.1038/srep29453

**Published:** 2016-07-11

**Authors:** X.-Y. Chang, D.-L. Deng, X.-X. Yuan, P.-Y. Hou, Y.-Y. Huang, L.-M. Duan

**Affiliations:** 1 Center for Quantum Information, IIIS, Tsinghua University, Beijing 100084, PR China; 2 Department of Physics, University of Michigan, Ann Arbor, Michigan 48109, USA

## Abstract

To construct a quantum network with many end users, it is critical to have a cost-efficient way to distribute entanglement over different network ends. We demonstrate an entanglement access network, where the expensive resource, the entangled photon source at the telecom wavelength and the core communication channel, is shared by many end users. Using this cost-efficient entanglement access network, we report experimental demonstration of a secure multiparty computation protocol, the privacy-preserving secure sum problem, based on the network quantum cryptography.

The peculiar quantum correlation, entanglement, provides the crucial resource for quantum information processing^1^,^2^,^3^. Generation of remote entanglement is a key step for quantum communication and distributed computation^4^,^5^. Distribution of entanglement in a multiple-end quantum network is costly through the point-to-point protocol as one needs to establish an entanglement source and a quantum communication channel between each end^4^,^5^,^6^,^7^,^8^,^9^,^10^. We demonstrate a cost-efficient entanglement access network where multiple end users share the same entanglement source and the core communication channel. We verify entanglement and quantum nonlocality between different end users. Using this entanglement access network, we report the experimental demonstration of a secure multi-party computation protocol where several end users compute cooperatively to solve a joint problem without revealing the information of their actual inputs^11^,^12^,^13^. This demonstration opens up the prospect of applying the cost-efficient entanglement access network for achieving secure multi-party computation that protects the privacy of end users.

With popularity of the internet, distributed computation becomes increasingly more important, where a number of end users need to work cooperatively to solve a common problem. The answer to the problem typically depends on the inputs of all the end users, which need to be communicated in the network. At the same time, the users need to protect their privacy and do not want to reveal their information to the others. Secure multi-party computation is a branch of cryptography and computer science that studies this kind of problems^11^,^12^,^13^. A well-known primitive example of this field is the millionaire problem, first introduced by Yao^11^, in which two millionaires want to know which of them is richer without revealing their actual wealth. Secure multi-party computation has many applications in e-commerce and data mining where people need to compare numbers which are confidential^11^,^12^,^13^. Classically, the secure multi-party computation protocols typically rely on computationally hard mathematical problems, which require specific assumptions and are subject to security loopholes in particular under attack by a quantum computer^13^.

In this paper, we demonstrate an entanglement access network which offers an alternative route for secure multi-party computation based on the network quantum cryptography. Quantum access network is a concept introduced in a recent paper where the expensive quantum resource is shared by many end users^8^,^9^. For quantum key distribution (QKD) based on the BB84 protocol^14^, the photon detector is the relatively expensive part of its implementation and thus shared in the quantum access network demonstrated in ref. 8. Here, we realize an entanglement access network to efficiently distribute entanglement between network end users. In this network, the entanglement source is the most expensive part of the implementation and thus shared between many end users. Entanglement provides the crucial quantum resource for achieving device-independent quantum cryptography, the most secure way for cryptographic communication^15^,^16^,^17^,^18^,^19^,^20^. Entanglement is also the critical resource for certified generation of shared randomness^21^, and for quantum communication and multi-party computation through the entanglement-based schemes^3^,^22^. We demonstrate an entanglement access network which efficiently distributes entanglement between a number of end users connected through coiled fibers of more than 20 km length by sharing a single entangled photon source at the telecom frequency. By use of this entanglement access network, we report experimental demonstration of a secure multi-party computation protocol, the secure sum problem, in which several millionaires want to know how much money they have in total but none of them is willing to reveal his wealth to others^11^,^12^. This demonstration shows that the entanglement access network provides a cost-efficient way to realize a quantum network with shared resource, opening up the prospect for its applications in secure multi-party cryptography and distributed quantum computation.

## Results

In our experiment, we first report a proof-of-principle demonstration of an entanglement-based network QKD scheme and then use this network QKD setup to realize a multi-party secure-sum computation protocol. In demonstration of the entanglement-based network QKD protocol, we share the same entangled photon source and divide each side into 8 parties with a 1 × 8 optical switch. This makes a 8 × 8 entanglement access network where each party of one side shares entanglement with any party on the other side. By choosing two parties from each side and detecting the photon polarization along three complementary directions, we explicitly demonstrate Bell inequality violation for different pairs of parties and a 4 × 4 entanglement-based QKD network. We then use this 4 × 4 QKD network to realize a four-party secure-sum protocol, where the four agents calculate their total wealth while keeping privacy of their individual wealth. Compared with the QKD network implemented through the BB84 protocol and the wavelength-division multiplexing device^23^,^24^, the implementation here has a much lower key exchange rate as the generation rate of entangled photons is much slower than the emission rate of weak coherent pulses used in the BB84 protocol. However, the entanglement-based QKD scheme could offer enhanced security^2^,^17^,^25^,^26^. In particular, it is a step towards eventual realization of the device-independent quantum cryptography^17^,^18^,^19^, the most secure way of communication, although implementation of the latter requires the very challenging condition to close all the loopholes in the Bell inequality test, which is not achieved in this experiment.

**Figure 1:**
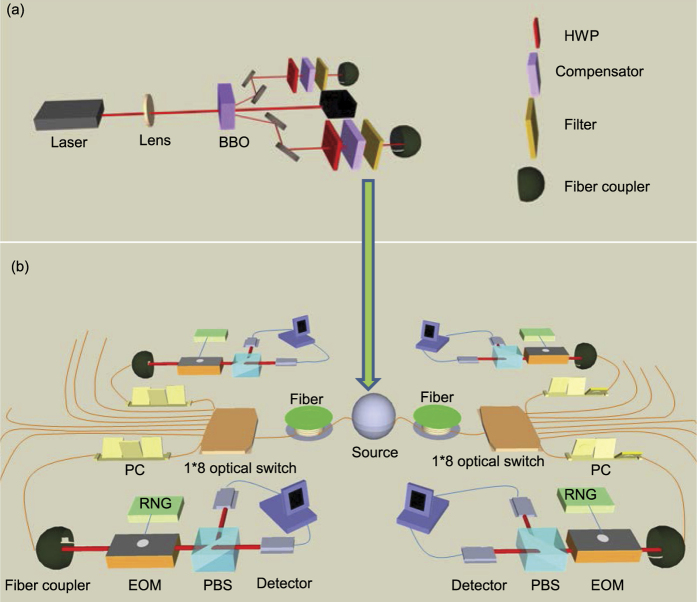
The experimental setup for implementation of an entanglement access network. (**a**) The setup to generate the polarized entangled photons at the telecom wavelength. The ultrafast pulse at the 775 nm wavelength from the Ti:Sapphire laser is *H*-polarized and focused by lens onto the BBO crystal cut in the Type-II phase-matching condition, generating polarized entangled photons at the 1550 nm wavelength. To ensure entanglement, we use a compensator made by another BBO crystal at each output, which compensates the temporal walk-off between the *H* and *V* polarized photons in the nonlinear crystal. The output photons are filtered by 3 nm interference filters and then coupled into long-distance coiled optical fibers with a fiber coupler. The half-wave plates (HWP) are used for alignment of the polarization axes before and after the fibers. (**b**) The setup to construct an entanglement access network, where the entanglement source and the core communication channel are shared by many end users. Computer controlled optical switches are used to distribute the entangled photons to different end users. Our experiment demonstrates an entanglement access network with up to 8 × 8 end users, where the two sides are separated by a fiber about 20 km. Among the end users, we have verified entanglement and quantum nonlocality between four pairs of them and used the shared entanglement to demonstrate the four-party secure sum protocol as an example to illustrate its application for implementation of secure multi-party computation.

The entangled photon source used in this experiment is shown in Fig. 1(a), which is generated by a type-II BBO crystal pumped by ultrafast laser pulses (with the pulse duration less than 150 fs and a pulse repetition rate of 76 MHz) at the wavelength of 775 nm from a Ti:Sapphire laser. The spontaneous parametric down conversion in the BBO crystal produces entangled photon pairs at the telecom wavelength of 1550 nm, which, in the ideal case, are in the polarization entangled state 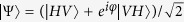, where |*H*〉 and |*V*〉 denote respectively the horizontal and the vertical polarization state and *φ* is a controllable phase^27^. We have verified that the experimentally generated state *ρ* has an entanglement fidelity *F*_*s*_ of (95.12 ± 0.25)% with respect to this ideal state |Ψ〉 through the quantum state tomography^28^. The coincidence count rate of the photon pairs is 710 per second for this source. This corresponds to an average pair number of 0.93 × 10^−5^ per pump pulse registered by the coincidence circuit. The small pair number here includes contribution of the low detection efficiency (~10% for each detector used in our experiment) at the telecom wavelength.

The entangled photons at the telecom frequency are coupled into coiled optical fibers representing the core communication channel with the total distance about 20 km. The output photons from the fibers are fed into a 1 × 8 optical switch (Dicon MS2-1X8-I2C-15-9/TB-FC/APC-1, with the insertion loss of 0.8 dB max) at each side (called Alice’s and Bob’s side) which is electrically controlled by the computer to deliver the photons to one of the eight output ports according to the electric control signal. This forms an entanglement access network with 8 × 8 possible choices of pairs of end users who can share entangled photons with the entanglement distance more than 20 km, as shown in Fig. 1(b). All the end users share the same entanglement source and the core communication channel. The polarization states of photons are subject to tension and temperature dependent rotations in the optical fibers which are carefully compensated afterwards through the fiber polarization controllers. In our experiment, the polarization entanglement is stable for more than 24 hours under a stable room-temperature environment. In the field experiment with longer communication distance, one may use the polarization locking technique reported in recent experiments to improve the stability of polarization entangled states^26^.

**Figure 2:**
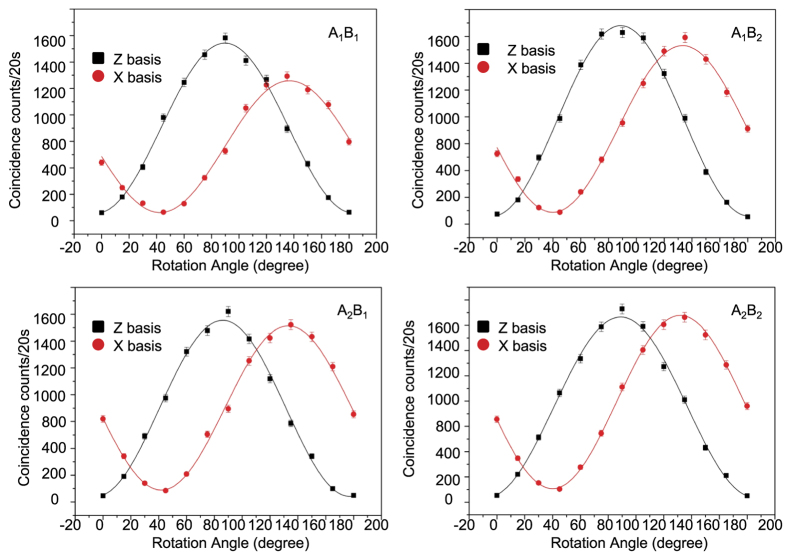
The entanglement demonstrated between different pairs of the end users. The figures show the coincidence counts when we fix the rotation angle of the electric optical modulator (EOM) of one end at 0° (for the Z-basis) or 45° (for the X-basis) while rotating the angle of the EOM at the other end. Different sub-figures correspond to different pairs of parties (*A*_1_ and *B*_1_, *A*_1_ and *B*_2_, *A*_2_ and *B*_1_, *A*_2_ and *B*_2_). The visibilities of the oscillations in the X and the Z bases together bound the entanglement fidelity of the photonic states shared between the corresponding parties.

To demonstrate entanglement between different end users, we randomly choose two users *A*_1_ and *A*_2_ at Alice’s side and two users *B*_1_ and *B*_2_ at Bob’s side. For the four pairs of end users *A*_*i*_*B*_*j*_ (*i*, *j* = 1, 2), we measure their entanglement by detecting the photon coincidences in different polarization bases. To rotate the polarization basis, we use a fast switchable electric optical modulator (EOM, Thorlabs EO-AM-NR-C3), which induces a polarization transformation |*H*〉→cos *θ* |*H*〉 − *i* sin *θ* |*V*〉 and |*V*〉→−*i* sin *θ* |*H*〉 + cos *θ* |*V*〉 with the angle *θ* determined by the computer controlled electric signal (see the Methods for control of the EOM). The output *H* and *V* polarized photons are split by a polarization beam splitter (PBS) and then detected through single photon counters. In the Z (or X) basis detection, we register the photon coincidence counts by fixing the angle *θ*_*A*_ of *A*_*i*_ at 0° (or 45°) and rotating the angle *θ*_*B*_ of *B*_*j*_. The resulting coincidence counts as functions of the angle *θ*_*B*_ are shown in Fig. 2 for different pairs *A*_*i*_*B*_*j*_. The big contrast of these oscillations is a clear demonstration of quantum entanglement. Quantitatively, we calculate the visibilities of these oscillation curves *V*_*z*_ and *V*_*x*_ in the Z and the X basis, respectively, and the entanglement fidelity *F*_*e*_ is then bounded by *F*_*e*_ ≥ (*V*_*z*_ + *V*_*x*_)/2 (see the Methods for definition of the visibilities and derivation of this criterion^29^). The visibilities and the corresponding fidelity bounds are listed in Table 1. All the pairs have the entanglement fidelity higher than 90%. The small decrease of the entanglement fidelity compared with the fidelity *F*_*s*_ of the entangled photon source is due to the imperfection in compensation of the polarization rotation in the optical fibers.

The shared entanglement allows demonstration of quantum key distribution between any pair of the end users in this network using the Ekert protocol^2^. For this purpose, we need to randomly choose the angle *θ*_*A*_ from the set {0°, 22.5°, 45°} and *θ*_*B*_ from the set {22.5°, 45°, 67.5°}, and record the individual measurement outcomes for each coincidence count^25^. From the counts for the angles *θ*_*A*_ = {0°, 45°} and *θ*_*B*_ = {22.5°, 67.5°}, we calculate the expectation value of the Clauser-Horne-Shimony-Holt (CHSH) observable 〈*S*〉 = 〈0°, 22.5°〉 + 〈45°, 22.5°〉 + 〈45°, 67.5°〉 − 〈0°, 67.5°〉^30^, where 〈*θ*_*A*_, *θ*_*B*_〉 denotes the photon coincidence counts with *θ*_*A*_, *θ*_*B*_ at the specified values. The CHSH values are listed in Table 1 for different pairs *A*_*i*_, *B*_*j*_. The CHSH inequality 〈*S*〉 ≤ 2 for any classical correlation is clearly violated, demonstrating quantum nonlocality. The significant violation of the CHSH inequality for each pair of the end users is a guarantee of the security of the entangled-based QKD protocol^15^,^16^,^17^,^18^,^19^,^20^. To generate quantum key, the measurement outcomes at *θ*_*A*_ = *θ*_*B*_ = {22.5°, 45°} are kept, which yield the sifted keys^25^. For each pair of the parties, we obtain raw keys of 1.2 × 10^4^ bits with an integration time of 1240 seconds. We randomly choose 20% of the keys to estimate the quantum bit error rate (QBER). For our data, the QBER *γ* is about 5%, smaller than the security threshold of 11%^20^. We use the low density parity check (LDPC) code^31^,^32^ to do the error correction for the sifted keys, which is more efficient compared with the conventional CASCADE protocol^25^. We obtain error-free shared keys of about 8 × 10^3^ bits, which are then purified by the privacy amplification protocol via a universal-2-class hash function^33^, yielding shared secret final keys of 1800 bits. The final key rate is about 1.5 bits per second. The residual information available to any potential eavesdroppers is reduced by a factor of 2^−300^ during the privacy amplification and thus much less than one bit.

We now use the entanglement access network to demonstrate a secure multi-party computation protocol: the privacy-preserving secure sum problem^12^. The problem can be illustrated with the following example: assume several millionaires want to know how much money they have in total, but none of them want to reveal his actual wealth to others. To be concrete, we consider the case of four parties *A*_1_, *A*_2_, *B*_1_, *B*_2_. Denote by *a*_1_, *a*_2_, *b*_1_ and *b*_2_ the input from *A*_1_, *A*_2_, *B*_1_, *B*_2_, respectively. We want to calculate the sum *T* = *a*_1_ + *a*_2_ + *b*_1_ + *b*_2_ without revealing the inputs *a*_1_, *a*_2_, *b*_1_, *b*_2_. The quantum protocol to accomplish this task using the entanglement access network goes as follows:

*Step 1*.—Using the entanglement-based network QKD, *A*_1_ and *B*_1_, *B*_1_ and *A*_2_, *A*_2_ and *B*_2_, and *B*_2_ and *A*_1_ share random keys of *n* bits denoted by 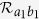, 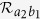, 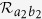, and 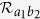, respectively. The number of bits *n* is taken to be at least as large as the estimated number of bits for the sum *T*.

*Step 2*.—*A*_1_ calculates 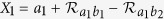 and publicly announces *X*_1_ to others; *B*_1_ calculates 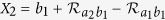 and publicly announces *X*_2_ to others; *A*_2_ calculates 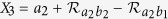 and publicly announces *X*_3_ to others; *B*_2_ calculates 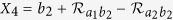 and publicly announces *X*_4_ to others.

*Step 3*.—All of them know the sum *T* by calculating *T* = *X*_1_ + *X*_2_ + *X*_3_ + *X*_4_. At the same time, the inputs *a*_1_, *a*_2_, *b*_1_, *b*_2_ remain confidential to the other parties as the public information *X*_*i*_ (*i* = 1, 2, 3, 4) has no correlation with the inputs due to the one-time pad theorem. For security of this secure sum protocol, we have assumed that different parties do not collaborate to steal the input information of the other parties. With the sum *T*, when three parties collaborate, it is always possible to reveal the input information of the fourth party. When two parties collaborate, whether they can reveal the information of the other two parties depends on whether these two parties share a random key in this protocol. If they share a random key (such as *A*_1_ and *B*_1_), they cannot conspire to reveal the wealth of the other party. For instance, even if *A*_1_ and *B*_1_ cooperate, they cannot reveal the wealth of *A*_2_ or *B*_2_ because they do not have the information of 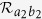, which is required to read out *a*_2_ or *b*_2_. On the other hand, if the two parties (such as *B*_1_ and *B*_2_) do not share a random key, they are able to conspire to reveal the wealth of the other two parties. The above protocol can be extended to more than four parties, and in general we need the assumption that different parties do not cooperate to simplify the security analysis.

**Figure 3:**
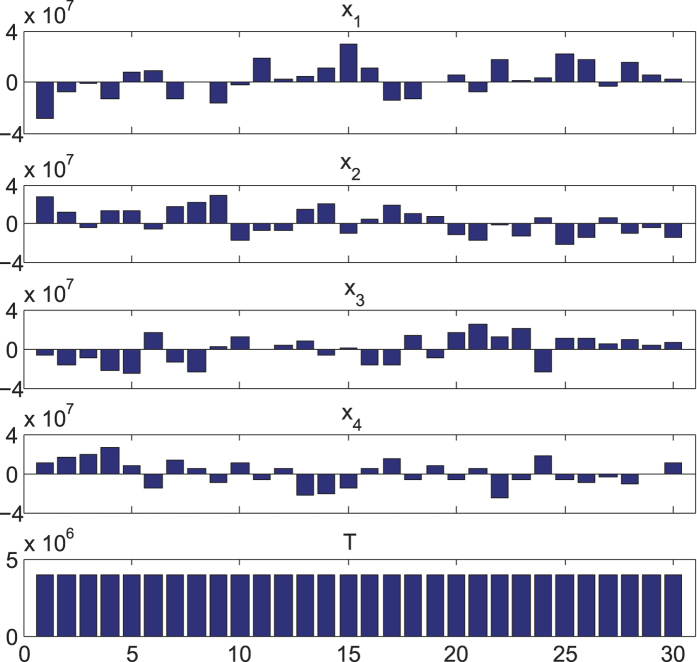
The experimental demonstration of the four-party secure-sum protocol through the entanglement access network. The top four sub-figures show the publically announced data from each of the four parties for 30 rounds of experiments for demonstration of the same secure-sum problem with the same input from each party. The randomly distributed data over different experimental runs is an indication that the announced data reveal no information of the input from each party. The last sub-figure shows the secure sum calculated by each party from the publically announced data, which is identical for different experimental runs and always equal to the true value of the sum for the underlying problem.

In the experimental demonstration, as an example, we take randomly generated numbers *a*_1_ = 55406, *b*_1_ = 116559, *a*_2_ = 988150 and *b*_2_ = 2839885. We run the above implementation of the secure sum protocol 30 times, and for each time we use different keys 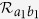, 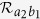, 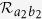, 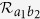 with the number of bits *n* = 25 bits to encode the public information *X*_1_, *X*_2_, *X*_3_, *X*_4_. For these 30 experimental runs (each experimental run is an implementation of the full QKD protocol that generates at least 25 bits of final keys 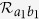, 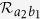, 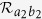, and 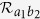 between the corresponding parties), the publicly announced numbers *X*_1_, *X*_2_, *X*_3_, *X*_4_ are shown in Fig. 3, which look completely random from trial to trial and reveal no information of the inputs *a*_1_, *a*_2_, *b*_1_, *b*_2_. However, for each run, their sum is always fixed to be 4 × 10^6^, which gives the correct calculation result for *T* = *a*_1_ + *a*_2_ + *b*_1_ + *b*_2_.

## Discussion

Similar to the quantum access network realized recently^8^, we expect that the entanglement access network demonstrated in this paper provides a source-efficient way for network cryptography and secure multi-party computation. In particular, the shared entanglement between each ends of the network opens up the possibility to realize device independent quantum cryptography^15^,^16^,^17^,^18^,^19^, which allows most secure communication by closing the security loopholes in conventional quantum cryptography^20^. Apart from the example demonstrated in this paper, the entanglement access network may allow realization of a number of secure multi-party computation problems^13^. The entanglement shared in the network could also find applications for certified generation of random numbers^21^ and for implementation of distributed quantum computation and multiparty quantum cryptography protocols^1^,^3^.

## Methods

### Control of the electric optical modulator

The electro-optic modulator (EOM) used in our experiment is a Pockels cell type modulator consisting of two lithium niobate crystals packaged in a compact housing with an RF input connector. Voltage applied across the crystal structure induces change in the indices of refraction (both ordinary and extraordinary), leading to an electric field dependent birefringence. An optical wave (with polarization components on both ordinary and extraordinary axes) will experience a change in polarization state after traversing the crystal, from the relative phase delay between these two orthogonal polarization components. The electro-optic crystal (EOM) thus acts as a wave-plate of variable rotation angle with the angle linearly dependent on the applied voltage.

In our experiment, the applied voltage is controlled by the random numbers generated from a computer program. The random numbers are fed into a FPGA (filed programmable gate array) board to generate the electric voltage signal. The voltage range from the FPGA board is only from 0–2 V, which is not large enough to induce a polarization rotation of the EOM corresponding to a quarter wave plate operation. The voltage for the latter is required to be 580 V, so we need to apply a voltage amplifier to the output signal of the FPGA board. The switching speed of the whole setup is at 500 Hz, which is limited by the response time of the voltage amplifier. As the recorded coincidence count rate for polarization detection is only about 70 per second at the receiver side, which is significantly less than the switching speed of the EOM device, we have an independent random setting for each pair of the photons that are recorded for quantum keys.

### Entanglement criterion based on the visibilities

In ref. 29, an entanglement criterion has been derived based on detection of correlations in two complementary bases. Here, we write this criterion into a more compact form in terms of the visibilities. For two correlated photons (or any qubits) detected in two complementary polarization bases *Z* = {|*H*〉, |*V*〉} and *X* = {|+〉, |−〉}, where 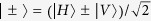, ref. 29 derived that the entanglement fidelity *F*_*e*_ (the overlap with a maximally entangled state) of a mixed state *ρ* is bounded by


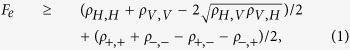


where *ρ*_*α*,*β*_ denotes the diagonal matrix elements (correlations) with the first (second) photon in *α* (*β*) polarization state. When we detect the visibility in the *Z* basis, the maximum and the minimum of the correlations are given by *ρ*_*H*,*H*_ (*ρ*_*V*,*V*_) and *ρ*_*H*,*V*_ (*ρ*_*V*,*H*_), respectively, when the first photon is fixed at *H* (*V*) polarization. So we define the average visibility in the *Z* basis as *V*_*z*_ = (*ρ*_*H*,*H*_ + *ρ*_*V*,*V*_ − *ρ*_*H*,*V*_ − *ρ*_*V*,*H*_)/(*ρ*_*H*,*H*_ + *ρ*_*V*,*V*_ + *ρ*_*H*,*V*_ + *ρ*_*V*,*H*_). The denominator of *V*_*z*_ is simply 1 because of normalization. Similarly, we have *V*_*x*_ = *ρ*_+,+_ + *ρ*_−,−_ − *ρ*_+,−_ − *ρ*_−,+_. Using the inequality that 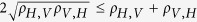, we write the bound for *F*_*e*_ as


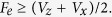


The entanglement fidelity *F*_*e*_ > 1/2 is a criterion for genuine entanglement^29^.

## Additional Information

**How to cite this article**: Chang, X.-Y. *et al*. Experimental realization of an entanglement access network and secure multi-party computation. *Sci. Rep.* **6**, 29453; doi: 10.1038/srep29453 (2016).

## Figures and Tables

**Table 1:** The experimental data to show entanglement and quantum nonlocality shared between different parties.

	Z basis visibility	X basis visibility	Fidelity bound	CHSH Value
*A*_1_*B*_1_	(91.68 ± 0.21)%	(88.76 ± 0.32)%	(90.22 ± 0.26)%	2.428 ± 0.020
*A*_1_*B*_2_	(92.59 ± 0.19)%	(88.11 ± 0.30)%	(90.35 ± 0.24)%	2.449 ± 0.019
*A*_2_*B*_1_	(94.10 ± 0.18)%	(87.91 ± 0.36)%	(91.01 ± 0.27)%	2.453 ± 0.020
*A*_2_*B*_2_	(93.54 ± 0.16)%	(86.73 ± 0.32)%	(90.14 ± 0.24)%	2.458 ± 0.021

The table lists the oscillation visibilities of the coincidence counts in the Z and the X bases, whose average gives a lower bound to the entanglement fidelity (the fourth column). The error bars are obtained by assuming a Poissionian distribution for the photon counts and propagated from the measured coincidence counts to the quantities listed in the table through exact Monte Carlo simulation. The last column of the table shows the measured value for the CHSH observable, and a larger-than-2 value indicates violation of the Bell inequality, demonstrating quantum nonlocality shared between the corresponding parties.
